# Parameters Optimization for Magnetic Resonance Coupling Wireless Power Transmission

**DOI:** 10.1155/2014/321203

**Published:** 2014-05-13

**Authors:** Changsheng Li, He Zhang, Xiaohua Jiang

**Affiliations:** ZNDY of Ministerial Key Laboratory, Nanjing University of Science and Technology, Nanjing 210094, China

## Abstract

Taking maximum power transmission and power stable transmission as research objectives, optimal design for the wireless power transmission system based on magnetic resonance coupling is carried out in this paper. Firstly, based on the mutual coupling model, mathematical expressions of optimal coupling coefficients for the maximum power transmission target are deduced. Whereafter, methods of enhancing power transmission stability based on parameters optimal design are investigated. It is found that the sensitivity of the load power to the transmission parameters can be reduced and the power transmission stability can be enhanced by improving the system resonance frequency or coupling coefficient between the driving/pick-up coil and the transmission/receiving coil. Experiment results are well conformed to the theoretical analysis conclusions.

## 1. Introduction

Wireless power transmission technology using magnetic resonance coupling, offering wireless mid-range power transmission, was originally proposed by a research group led by Marin Soljacic from MIT in 2007. They used a self-made wireless power device to power a 60-watt bulb from a distance of 2 m, with an efficiency of 40–50% [1]. The appearance of this technology breaks the traditional model of electromagnetic induction transmission for which efficiency strictly depended on the coupling coefficient of coils; wireless power transmission distances were extended from the millimeter to the meter range [2–7]. This represented a breakthrough in wireless power transmission technology.

With the development and application of wireless power transmission technology based on magnetic resonance coupling, parameters optimal design and optimal control for the power transmission process have become the focus of research [8–16]. To maximize transmission power or transmission efficiency, [9–11] analyzed the influence laws of operating parameters on the transmission performance, optimized load value, coil size, and quality factor of the system. However, with coupling coefficients between coils, as key parameters in system design, no optimal conclusion was drawn. Furthermore, influence laws of operating parameters on the transmission stability were not investigated. On the other hand, although magnetic resonance has significant advantage in transmission distance compared with electromagnetic induction, this technology has intrinsic limitation as the load absorption power is sensitive to variations in the operating parameters, and small differences in operating and resonance frequency will reduce transmission performance significantly. To solve this problem, an optimal control method of frequency-adaptive adjustment was proposed in [12, 13]. By applying modules for state detection, RF communication, and tracking adjustment in the transmission and receiving terminals, combined with optimization control algorithm, this method detects the system's working state in real time and dynamically adjusts the system working at the optimum state. This method is a good solution for application when transmission and receiving terminals are in a static or quasi-static state. However, as this method needs extra circuit modules in hardware and calculating time in software, it is unsuitable for situations with strict requirements in space and time. A typical example is high-speed dynamic cartridge link setting for projectiles in a weapon system. In terms of space, there has been strictly space limitation to accommodate electronic devices in projectiles. In terms of time, there is no time to adjust to and there is relative high-speed movement between transmission and receiving terminals (for a type of projectile, the setting rate is 5000 bullets/min). One possible solution could be to improve power transmission stability and reduce the sensitivity of power transmission performance to the variations in the operating parameters by optimal parameter design.

Based on the circuit model of magnetic resonance system that has been established in [17], using the theoretical analysis method of impedance mapping, the coupling coefficients of coils are optimized to maximize power transmission. Then, methods of improving power transmission stability and decreasing transmission performance sensitivity to variations in operating parameters are discussed.

## 2. Circuit Model

**Figure 1:**
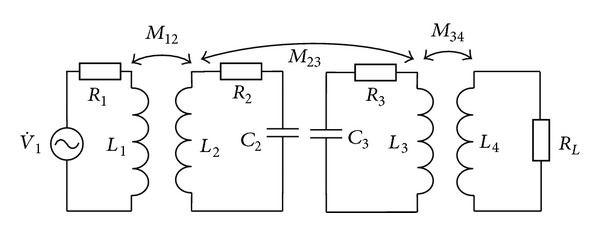
Equivalent circuit model of a resonant system.

The equivalent circuit model of this resonant system, based on mutual inductance theory, is shown in Figure 1 [17]. There, *L*_1_, *L*_2_, *L*_3_, and *L*_4_ are the self-inductance of the driving, transmission, receiving, and pick-up coils, respectively, *C*_2_ and *C*_3_ the respective resonance capacitances of the transmission and receiving coils, *R*_2_ and *R*_3_ the equivalent resistances of the transmission and receiving coils, *R*_1_ the sum of the power amplifier output resistance and the equivalent resistance of the driving coil, *R*_*L*_ the equivalent load of the system including the resistance of the pick-up coil, ${\dot{V}}_{1}$ the excitation voltage source, and *M*_*ij*_ and *k*_*ij*_ the respective mutual inductance and coupling coefficient of any pair of coils, with $M_{ij}=k_{ij}\sqrt{L_{i}L_{j}}$ and 0 ≤ *k*_*ij*_ ≤ 1.

For a resonant system, the transmission performance is not always increasing with the decreasing of separation between coils, which has been proved by experiment [9]. There is an optimal value for the coupling coefficient, at which the load gets the maximum power. In order to avoid mathematical calculation of the four equations established according the Kirchhoff's law, when solving the optimal values of coupling coefficient *k*_12_, *k*_23_, and *k*_34_, the analysis method of impedance mapping is adopted in this paper [18]. Through this method, the electric parameters of coils are mapped into the adjacent coils, and the four-coil system can be equivalent to a double-coil or a single-coil system, which will significantly reduce the difficulty of mathematical analysis.

The electric parameters of driving and pick-up coils are mapped into the transmission and receiving coils, respectively, and the results are as follows:

(1) Zf1=(ωM12)2R1+jωL1=R1′+1jωC1′,V˙2=jωM12R1+jωL1V˙1,Zf4=(ωM34)2RL+jωL4=RL′+1jωC4′.

There,

(2) $$\begin{array}{c} C_{1}^{\prime }=\frac{R_{1}^{2}+{\left(\omega L_{1}\right)}^{2}}{\omega^{2}L_{1}{\left(\omega M_{12}\right)}^{2}},\;\;R_{1}^{\prime }=\frac{R_{1}{\left(\omega M_{12}\right)}^{2}}{R_{1}^{2}+{\left(\omega L_{1}\right)}^{2}},\\ C_{4}^{\prime }=\frac{R_{L}^{2}+{\left(\omega L_{4}\right)}^{2}}{\omega^{2}L_{4}{\left(\omega M_{34}\right)}^{2}},\;\;\\ R_{L}^{\prime }=\frac{R_{L}{\left(\omega M_{34}\right)}^{2}}{R_{L}^{2}+{\left(\omega L_{4}\right)}^{2}},\;\;R_{22}=R_{1}^{\prime }+R_{2},\\ X_{22}=\omega L_{2}-\frac{C_{1}^{\prime }+C_{2}}{\omega {C^{\prime }}_{1}C_{2}},\;\;R_{33}=R_{3}+R_{L}^{\prime },\\ X_{33}=\omega L_{3}-\frac{C_{3}+C_{4}^{\prime }}{\omega C_{3}C_{4}^{\prime }}. \end{array}$$

**Figure 2:**
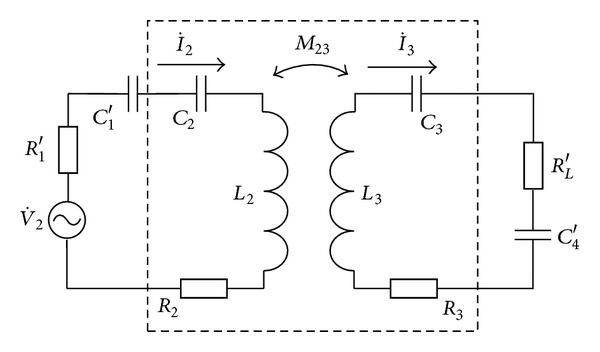
Double-coil equivalent circuit of a resonant system.

So, Figure 1 can be equaled to Figure 2.

From Figure 2, the transmission power of system can be written as

(3) P0=(0.5V2m2RL′ω2M232(R332+X332))×((R22+R33ω2M232R332+X332)2   +(X22−X33ω2M232R332+X332)2)−1.

By solving ∂*P*_0_/∂*k*_23_ = 0, the optimal coupling coefficient *k*_23_ for maximum power transmission can be obtained as follows:

(4) $$\begin{array}{c} k_{23\text{-opt}}=\frac{1}{\sqrt{L_{2}L_{3}}}{\left[\frac{\left(R_{22}^{2}+X_{22}^{2}\right){\left(R_{33}^{2}+X_{33}^{2}\right)}^{2}}{R_{33}^{2}\omega_{r}^{4}+X_{33}^{2}\omega_{r}^{4}}\right]}^{0.25}. \end{array}$$

When calculating optimal value of *k*_34_, in order to reduce the mathematical calculation difficulty for secondary mapping, (3) can be simplified. Under the resonant condition, there is *X*_22_ ≈ *X*_33_ ≈ 0. Therefore, optimal coupling coefficient *k*_34_ can be derived from (3) as

(5) $$\begin{array}{c} k_{34\text{-opt}}=\sqrt{\frac{\left(R_{22}R_{3}+\omega_{r}^{2}M_{23}^{2}\right)\left(R_{L}^{2}+\omega_{r}^{2}L_{4}^{2}\right)}{R_{22}R_{L}\omega_{r}^{2}L_{3}L_{4}}}. \end{array}$$

In (4) and (5), *ω*_*r*_ is the self-resonating angular frequency of the transmission and receiving coils under the influence of the drive and pick-up coils, respectively, when the system is operating at undercoupled state. By solving *X*_22_ ≈ *X*_33_ ≈ 0, *ω*_*r*_ can be expressed as follows:

(6) ωr≈([(L12+R12L2C2)2−4k122L12R12L2C2   +L12−R12L2C2]0.5) ×([2(1−k122)L12L2C2]0.5)−1≈([(L42+R42L3C3)2−4k342L42R42L3C3   +L42−R42L3C3]0.5) ×([2(1−k342)L42L3C3]0.5)−1.

**Figure 3:**
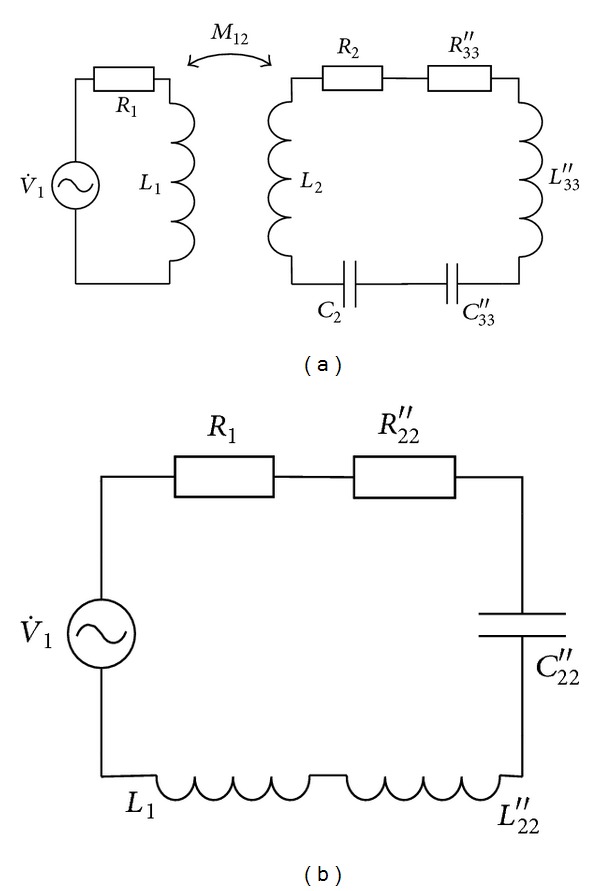
Mapping process for the resonant system.

For the solution of the optimal value *k*_12_, the electric parameters of the pick-up coil, the receiving coil, and the transmission coil are mapped into the adjacent loops in turn, and the resonant system can be equivalent to a single-coil system eventually. The mapping process is shown in Figure 3.

In Figure 3(a),

(7) $$\begin{array}{c} R_{33}^{\prime \prime }=\frac{R_{33}{\left(\omega M_{23}\right)}^{2}}{R_{33}^{2}+X_{33}^{2}},\;\;L_{33}^{\prime \prime }=\frac{M_{23}^{2}}{C_{33}\left(R_{33}^{2}+X_{33}^{2}\right)},\\ C_{33}^{\prime \prime }=\frac{R_{33}^{2}+X_{33}^{2}}{L_{3}\omega^{4}M_{23}^{2}},\;\;C_{33}=\frac{C_{3}C_{4}^{\prime }}{C_{3}+C_{4}^{\prime }}. \end{array}$$

In Figure 3(b),

(8) $$\begin{array}{c} R_{22}^{\prime \prime }=\frac{R_{222}{\left(\omega M_{12}\right)}^{2}}{R_{222}^{2}+X_{222}^{2}},\;\;L_{22}^{\prime \prime }=\frac{M_{12}^{2}}{C_{222}\left(R_{222}^{2}+X_{222}^{2}\right)},\\ C_{22}^{\prime \prime }=\frac{R_{222}^{2}+X_{222}^{2}}{L_{222}\omega^{4}M_{12}^{2}},\;\;R_{222}=R_{2}+R_{33}^{\prime \prime },\\ L_{222}=L_{2}+L_{33}^{\prime \prime },\;\;C_{222}=\frac{C_{2}C_{33}^{\prime \prime }}{C_{2}+C_{33}^{\prime \prime }},\\ X_{222}=\omega L_{222}-\frac{1}{\omega C_{222}}. \end{array}$$

The mapping resistance of load *R*_*L*_ at the driving coil is

(9) $$\begin{array}{c} R_{L}^{\prime \prime \prime }=\frac{R_{L}^{\prime }R_{33}^{\prime \prime }{\left(\omega M_{12}\right)}^{2}}{\left(R_{222}^{2}+X_{222}^{2}\right)R_{33}}. \end{array}$$

From Figure 3(b), the transmission power of system can be written as

(10) $$\begin{array}{c} P_{0}=\frac{0.5V_{1m}^{2}R_{L}^{\prime \prime \prime }}{{\left(R_{1}+{R_{22}^{\prime \prime }}_{}\right)}^{2}+{\left[\omega \left(L_{1}+L_{22}^{\prime \prime }\right)-\left(1/\omega C_{22}^{\prime \prime }\right)\right]}^{2}}. \end{array}$$

Optimal value *k*_12_ can be derived from (10) using calculus as follows:

(11) $$\begin{array}{c} k_{12\text{-opt}}=\sqrt{\frac{\sqrt{\varepsilon^{2}+4\delta \xi }-\varepsilon }{2\delta L_{1}L_{2}}}. \end{array}$$

In (11), *ε* = 2*R*_222_ *C*_222_^2^ *ω*_*r*_^2^ *R*_1_ *τ*, *δ* = 3*R*_222_^2^ *C*_222_^2^ *ω*_*r*_^4^ + *ς* ^2^, *ξ* = *σ* ^2^ + *C*_222_^2^ *R*_1_^2^ *τ* ^2^, *τ* = *R*_222_^2^ + *X*_222_^2^, and *ς* = *ω*_*r*_ − *L*_222_ *C*_222_ *ω*_*r*_^3^, *σ* = *ω*_*r*_ *L*_1_ *C*_222_ *τ*.

From (4), (5) and (11), the optimal values of coupling coefficients are closely related to the resonance angular frequency *ω*_*r*_. In the condition of undercoupling and near the critical coupling, *ω*_*r*_ can be approximated as a constant, as (6). But in the overcoupling region, the resonance frequency appears as splitting phenomena and the values vary sharply with increasing of *k*_23_. Therefore, the formulas developed in this paper can only be used in the condition of undercoupling and near the critical coupling. On the other hand, the significant advantage of resonance technology compared with electromagnetic induction technology is the farther distance of wireless transmission. Therefore, the optimal design for large distance (nonovercoupling region) has good practical engineering value. The coupling status of the resonant system is determined by *k*_23_; the value of the coupling coefficient *k*_23_ at critical coupling status is called critical coupling coefficient, denoted as *k*_*c*_. If *k*_23_ > *k*_*c*_, the coupling status is overcoupling, whereas if *k*_23_ < *k*_*c*_, the status is undercoupling. Referring the solving method of *k*_*c*_ to double-coil system in [19], from Figure 2, the critical coupling coefficient of this four-coil system can be written as

(12) $$\begin{array}{c} k_{c}={\left[\frac{{\left(R_{1}^{\prime }+R_{2}\right)}^{2}+{\left(R_{L}^{\prime }+R_{3}\right)}^{2}}{2\omega_{r}^{2}L_{2}^{2}}\right]}^{0.5}. \end{array}$$

**Figure 4:**
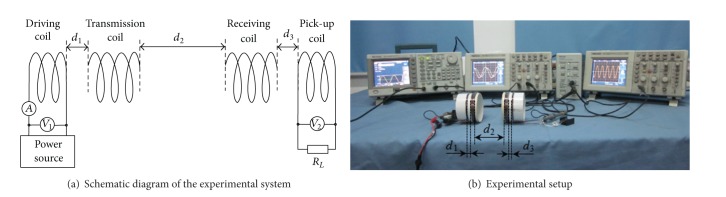
Experimental system.

To verify the above optimization theory, experimental analysis for a resonant system is performed. The experimental system is shown in Figure 4. The coil is 75 mm in diameter and is wound with 0.9 mm diameter enameled copper wire, and the load resistance is 50 Ω. The power source used in the experiment is a signal generator (Tektronix AFG3102, peak value of output voltage 5 V, and output impedance 50 Ω). Power measurements are performed using a current probe (Tektronix TCP312 with TCPA300) with oscilloscopes (Tektronix TDS2022). The transmission and the receiving terminals are placed coaxially and are able to be displaced along the axis. The separations between driving and transmission coils, transmission and receiving coils, and receiving and pick-up coils are denoted as *d*_1_, *d*_2_, and *d*_3_, respectively. The number of turns in driving and pick-up coils is 2 and in transmission and receiving coils is 5.

The key parameters of coils in Table 1 are measured by the LCR meter (HIOKI 3532-50).

**Figure 5:**
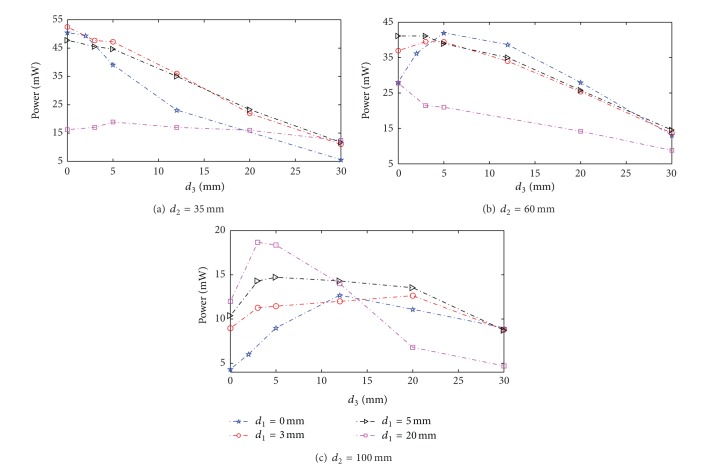
Variations in transmission power with separation between coils.

Figure 5 is the experiment curves of variations in transmission power with separation between coils. Optimal values of coupling coefficients are listed in Tables 2, 3, and 4.

From Tables 2–4, we can see that the optimal values obtained from theoretical calculation and experiment are well consistent. Error is mainly caused by the following two reasons: one is the resonance angular frequency taken in theoretical calculation that is an approximation as (6) and the other is the that separation between coils can only be changed step by step. The experimental results showed that the optimization formulas are correct and can be used to effectively optimize the power transmission characteristics in the nonovercoupling region for resonant systems.

## 3. Methods for Enhancing Power Transmission Stability

The power transmission performance of the resonant system is determined by the synthesis of different transmission parameters. Peak power output is acquired at the resonance frequency point, and the absorption power of the load declines sharply when the operating frequency deviates from the resonance frequency. In engineering applications, the transmission performance may decline for the differences in actual and design resonance frequency caused by fabrication process of coils, relative movement between transmission and receiving terminals, interference of environmental factors, and other reasons. The possible solution for this problem is to improve the pass bandwidth of the system to reduce the sensitivity of the transmission power to operating frequency variations by optimal design for parameters. That is to say, when the deviation in the operating frequency from the resonance frequency is small, even without frequency-adaptive adjustment, the system can still output high power with efficiency.

**Figure 6:**
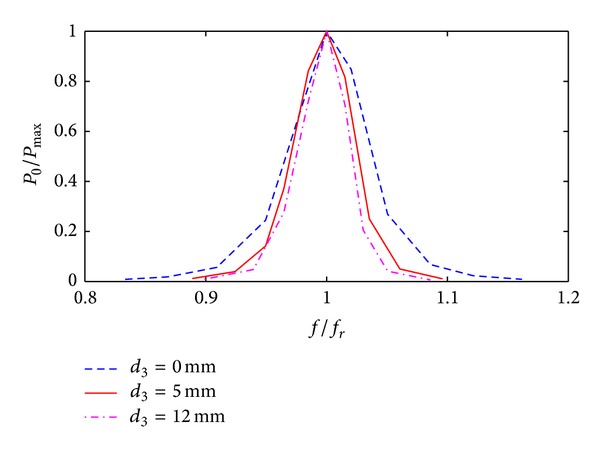
Variation of load power with frequency at various *d*_3_ values.

**Figure 7:**
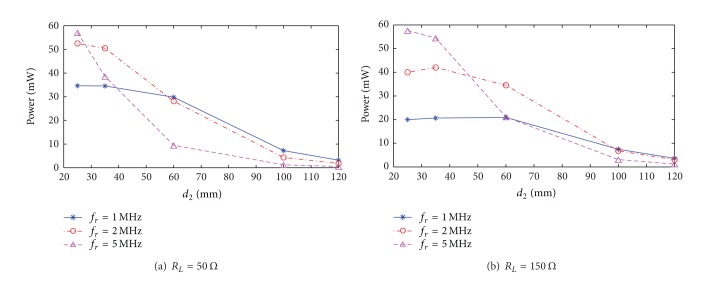
Variations in power transmission characteristics with various resonance frequencies.

**Figure 8:**
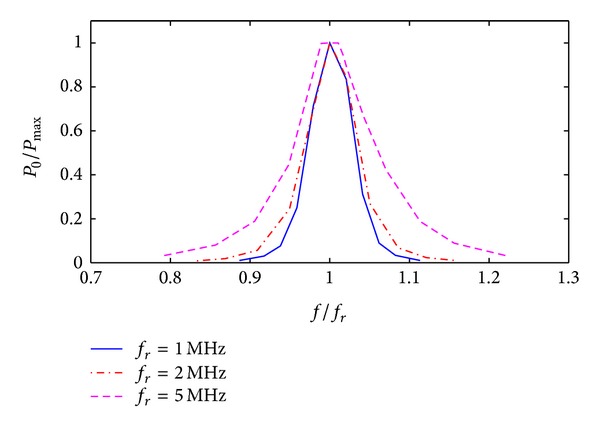
Variations in frequency response curve of load power with various resonance frequencies.

Simulation analysis and experimental results all show that, by improving the system resonance frequency or coupling coefficient *k*_12_, *k*_34_ can effectively improve the power transmission stability under conditions that affect less negatively other transmission performances. The following introduces these two methods and analyzes what negative influence these may introduce.

(*1) Increasing Values of the Coupling Coefficients k*_12_ *and k*_34_. The degree of sharpness in the frequency response curve of the load power reduces as the coupling coefficients *k*_12_ and *k*_34_ increase. By reducing the axial distance between the driving coil (pick-up coil) and the transmission coil (receiving coil), values of the coupling coefficients *k*_12_ and *k*_34_ can be increased. This will play an active role in reducing the degree of sharpness in the frequency response curve of the load power. On the other hand, from the angle of maximum power transmission, it is not the larger of coupling coefficients the better. By increasing *k*_12_, *k*_34_, to enhance power transmission stability, may reduce the amplitude of power transmission simultaneous. As illustrated by the experiment curves in Figure 5, transmission power may increase as *d*_1_ and *d*_3_ increase. This phenomenon is even more apparent when the value of *d*_2_ is larger.  Figure 6 shows the experimental curve of the normalized load power at various excitation frequencies settings *d*_1_ = 0 and *d*_2_ = 60 mm; *P*_max⁡_ is the load absorption power at resonance. Clearly, the system pass bandwidth increases 33 KHz when *d*_3_ is decreased from 12 mm to 0. 

(*2) Increasing the Resonance Frequency.* The resonance frequency is a key parameter in system design, whose value can be changed by adjusting the number of turns or the externally matched capacitance of the transmission and receiving coils. The degree of sharpness in the frequency response curve of load power reduces as resonance frequency increases. Therefore, enhancing power transmission stability can be obtained by properly reducing the value of the external matched capacitance. Transmission systems with a high resonance frequency can produce high power transmission over short distances. However, transmission performance attenuates fast as the separation between transmission and receiving terminals increases. Therefore, it is not appropriate for the wireless transmission of power over long distance. The attenuation of transmission power for systems with small resonance frequencies is relatively slow as the separation between transmission and receiving terminals increase. This is shown in Figure 7.  Figure 8 gives the normalized experimental curve of load power for various excitation frequencies at *d*_2_ = 60 mm and *R*_*L*_ = 50 Ω. It shows that when *f*_*r*_ = 5 MHz, the pass bandwidth is 236 KHz and 285 KHz wider than that at 2 MHz and 1 MHz, respectively. In addition, along with increasing the resonance frequency, difficulties of circuit implementing increase, and inversion losses in the driving circuit also increase. These factors will cause further degradation in total transmission efficiency.

To illustrate the effect of optimization, both Figures 6 and 8 show normalized curves of the resonant system with only a single resonance frequency. When the separation between transmission and receiving terminals is small and the system shows splitting in the resonance frequency, the optimization method is still applicable.

In summary, by appropriate readjustment of the transmission parameters, the sharpness in the frequency response curve of load power can be effectively reduced. Simultaneously, transmission power might be reduced when the power transmission stability is improved. In contrast, wireless power transmission technology based on magnetic resonance coupling relies on a strong coupling between transmission and receiving terminals to produce high efficiency power transmission. The transmission theory is established based on resonance, which in principle determines the sensitivity of the power transfer characteristics to the working frequency. Therefore, the sensitivity cannot be totally eliminated. During engineering-design stages, the transmission parameters should be set appropriately by accommodating with the transmission performance of the system.

## 4. Conclusion

In this paper, power transmission characteristics of magnetic resonance coupling wireless power transmission system are optimized. Based on the mutual coupling model of a resonant system, optimization formulas of coupling coefficient in the condition of maximum power transmission are deduced, and experimental results show that the optimization formulas are correct and can be used to effectively optimize power transmission characteristics in the nonovercoupling region. On the other hand, by improving the system resonance frequency or coupling coefficient *k*_12_, *k*_34_, the power transmission stability can be improved while power transmission performance sensitivity to variations in operating parameters can be decreased. The conclusions obtained in this paper will enrich the theory of wireless power transmission based on magnetic resonance and provide a reference for engineering applications.

## Figures and Tables

**Table 1:** Coil parameters.

Coils	Self-inductance (*μ*H)	Resistance (Ω)	Matched capacitance (nF)
Driving	0.89	0.14	0
Transmission	3.80	0.54	1.68
Receiving	3.64	0.54	1.85
Pick-up	0.86	0.14	0

**Table 2:** Theoretical and experimental values of *k*_12-opt_.

Experiment conditions		Theory	Experiment
*k*_23_ (*d*_2_ mm)	*k*_34_ (*d*_3_ mm)	*k*_12_	*k*_12_ (*d*_1_ mm)
0.101 (35)	0.600 (0)	0.451	0.414 (3)
0.019 (100)	0.414 (3)	0.179	0.176 (20)
0.050 (60)	0.414 (3)	0.320	0.367 (5)
0.153 (25)	0.353 (5)	1.000*	1.000*

*The separation between coils is as close as possible, the same meaning as in Table 3.

**Table 3:** Theoretical and experimental values of *k*_34-opt_.

Experiment conditions		Theory	Experiment
*k*_12_ (*d*_1_ mm)	*k*_23_ (*d*_2_ mm)	*k*_34_	*k*_34_ (*d*_3_ mm)
0.367 (5)	0.050 (60)	0.602	0.600 (0)
0.450 (3)	0.050 (60)	0.528	0.479 (2)
0.102 (30)	0.019 (100)	0.437	0.414 (3)
0.367 (5)	0.101 (35)	1.000*	1.000*

**Table 4:** Theoretical and experimental values of *k*_23-opt_.

Experiment conditions		Theory	Experiment
*k*_12_ (*d*_1_ mm)	*k*_34_ (*d*_3_ mm)	*k*_23_	*k*_23_ (*d*_2_ mm)
0.102 (30)	0.414 (3)	0.037	0.035 (73)
0.450 (3)	0.150 (20)	0.046	0.050 (60)
0.222 (12)	0.600 (0)	0.050	0.050 (60)
0.450 (3)	0.095 (30)	0.045	0.050 (60)

## References

[1] Kurs A, Karalis A, Moffatt R, Joannopoulos JD, Fisher P, Soljacic M (2007). Wireless power transfer via strongly coupled magnetic resonances. *Science*.

[2] Tucker CA, Warwick K, Holderbaum W (2013). A contribution to the wireless transmission of power. *International Journal of Electrical Power & Energy Systems*.

[3] Karalis A, Joannopoulos JD, Soljacic M (2008). Efficient wireless non-radiative mid-range energy transfer. *Annals of Physics*.

[4] Chan T, Chen C (2012). A primary side control method for wireless energy transmission system. *IEEE Transactions on Circuits and Systems I: Regular Papers*.

[5] Lee CK, Zhong WX, Hui SYR (2012). Effects of magnetic coupling of nonadjacent resonators on wireless power domino-resonator systems. *IEEE Transactions on Power Electronics*.

[6] Ahn D, Hong S (2013). Effect of coupling between multiple transmitters or multiple receivers on wireless power transfer. *IEEE Transactions on Industrial Electronics*.

[7] Li Y, Yang Q, Yan Z (2012). Characteristic of frequency in wireless power transfer system via magnetic resonance coupling. *Electric Machines and Control*.

[8] Wang J, Zhu Z, Li C, Huangfu J, Ran L (2013). PLL-based self-adaptive resonance tuning for a wireless-powered potentiometer. *IEEE Transactions on Circuits and Systems II: Express Briefs*.

[9] Kim Y-H, Kang S-Y, Lee M-L, Yu B-G, Zyung T Optimization of wireless power transmission through resonant coupling.

[10] Huh J, Lee W, Choi S, Cho G, Rim C (2013). Frequency-domain circuit model and analysis of coupled magnetic resonance systems. *Journal of Power Electronics*.

[11] Jonal O, Georgakopoulos SV, Tentzeris MM (2012). Optimal design parameters for wireless power transfer by resonance magnetic. *IEEE Antennas and Wireless Propagation Letters*.

[12] Sample AP, Meyer DA, Smith JR (2011). Analysis, experimental results, and range adaptation of magnetically coupled resonators for wireless power transfer. *IEEE Transactions on Industrial Electronics*.

[13] Kim NY, Kim KY, Kim CW (2012). Automated frequency tracking system for efficient mid-range magnetic resonance wireless power transfer. *Microwave and Optical Technology Letters*.

[14] Kim JW, Son H-C, Kim K-H, Park Y-J (2011). Efficiency analysis of magnetic resonance wireless power transfer with intermediate resonant coil. *IEEE Antennas and Wireless Propagation Letters*.

[15] Tan LL, Huang XL, Huang H, Zou Y, Li H (2011). Transfer efficiency optimal control of magnetic resonance coupled system of wireless power transfer based on frequency control. *Science China Technological Sciences*.

[16] Xue R, Cheng K, Je M (2013). High-efficiency wireless power transfer for biomedical implants by optimal resonant load transformation. *IEEE Transactions on Circuits and Systems I: Regular Papers*.

[17] Ko YY, Ho SL, Fu WN, Zhang X (2012). A novel hybrid resonator for wireless power delivery in bio-implantable devices. *IEEE Transactions OnMagnetics*.

[18] Huang J (2003). *Cirruits*.

[19] Niu WQ, Gu W, Chu JX, Shen AD (2012). Coupled-mode analysis of frequency splitting phenomena in CPT systems. *Electronics Letters*.

